# Socioeconomic inequalities in survival of children with acute lymphoblastic leukemia insured by social security in Mexico: a study of the 2007–2009 cohorts

**DOI:** 10.1186/s12939-019-0940-3

**Published:** 2019-03-04

**Authors:** Angélica Castro-Ríos, Hortensia Reyes-Morales, Blanca E. Pelcastre, Mario E. Rendón-Macías, Arturo Fajardo-Gutiérrez

**Affiliations:** 1 Unidad de Investigación en Epidemiología Clínica, Hospital de Pediatría, Centro Médico Nacional SXXI, Instituto Mexicano del Seguro Social, Avenida Cuauhtémoc 330, Col. Doctores, Ciudad de México, Mexico; 2 Centro de Investigación en Sistemas de Salud, Instituto Nacional de Salud Pública, Cuernavaca, Morelos Mexico; 3 Public Health Department, Universidad Panamericana, Ciudad de México, Mexico

**Keywords:** Social inequalities in health, Acute lymphoblastic leukemia, Cancer survival, Mexico, Social security, Social determinants in health

## Abstract

**Background:**

Although acute lymphoblastic leukemia (ALL) 5 years survival in minors has reached 90%, socioeconomic differences have been reported among and within countries. Within countries, the difference has been related to the socioeconomic status of the parents, even in the context of public health services with universal coverage. In Mexico, differences in the mortality of children with cancer have been reported among sociodemographic zones. The Instituto Mexicano del Seguro Social (IMSS), the country’s main social security institution, has reported socioeconomic differences in life expectancy within its affiliated population. Here, the socioeconomic inequalities in the survival of children (< 15 years old) enrolled in the IMSS were analyzed.

**Methods:**

Five-year survival data were analyzed in cohorts of patients diagnosed with ALL during the period 2007–2009 in the two IMSS networks of medical services that serve 7 states of the central region of Mexico. A Cox proportional risk model was developed and adjusted for the socioeconomic characteristics of family, community of residence and for the clinical characteristics of the children. The slope of socioeconomic inequality of the probability of dying within five years after the diagnosis of ALL was estimated.

**Results:**

For the 294 patients studied, the 5 years survival rate was 53.7%; the median survival was 4.06 years (4.9 years for standard-risk diagnosis; 2.5 years for high-risk diagnosis). The attrition rate was 12%. The Cox model showed that children who had been IMSS-insured for less than half their lives had more than double the risk of dying than those who had been insured for their entire lives.

**Conclusions:**

We did not find evidence of socioeconomic inequalities in the survival of children with ALL associated with family income, educational and occupational level of parents. However, we found a relevant gradient related social security protection: the longer children’s life insured by social security, the higher their probability of surviving ALL was. These results add evidence of the effectiveness of social security, as a mechanism of wealth redistribution and a promoter of social mobility. Extending these social security benefits to the entire Mexican population could promote better health outcomes.

**Electronic supplementary material:**

The online version of this article (10.1186/s12939-019-0940-3) contains supplementary material, which is available to authorized users.

## Background

Cancer is a major cause of death in children worldwide and the overall age-standardized incidence rate (ASR) in children (< 15 years old) is 140·6 per million person-years, and the most common cancers is leukaemia (46·4 per million) [1]. In Mexico the ASR for leukaemia is 61.6 [1] and for lymphoid leukaemia 50.7 per million [2] (~ 2500 new cases each year). [3]

In high-income countries, a sustained increase in the survival of children with acute lymphoblastic leukemia (ALL) has been observed, reaching 90% over the past decade [4–6].

However, a differentiated pattern in survival was observed according to socioeconomic level, not only between countries but also within countries. [7]

In Latin America in 2010–2014, Puerto Rico reported the highest survival (93%), followed by Costa Rica (80%) and Argentina (76%); Colombia, Brazil, Chile, and Perú reported a survival of less than 70%, and Ecuador and Mexico reported a survival of less than 60%. [4]

Within countries, disparities in the survival of children have also been reported and have been related to parental socioeconomic status. In a study of almost six million children in South Korea, Son et al. [8] reported that among children with cancer, the risk of dying among children whose parents were unemployed was 73% higher than that among those whose parents performed non-manual labor and 26% higher than that among those whose parents were manual laborers, whereas the risk of dying was 37.6% lower among children whose mothers had a university education than among those whose mothers had not finished high school.

Even in the context of public health services with universal coverage, differences in survival are associated with the socioeconomic conditions of the parents. In the United Kingdom, Lightfoot et al. [9] analyzed the inequalities in the survival of children with ALL according to the degree of marginalization of their place of residence, finding that children living in zones with higher marginalization had a 29% greater risk of death than did those living in less marginalized areas and that the risk was 12% higher in children of parents in occupations requiring lower qualifications than in children of parents in professional occupations.

In the Latin American context, in Brazil [10], a negative correlation was found between mortality due to ALL in children and the exclusion level of the province of residence (− 0.66 aprox).

In Mexico, a study by Escamilla et al. [11], reported on social inequality for children with cancer in the Mexican population and analyzed two decades (1990–2009) of data, reporting that the mortality from childhood cancer increased 28% during that period and that this increase was higher in the states of Mexico having high or very high marginalization. Specifically, for ALL, they observed an increase of ~ 50%—threefold higher than the mortality reported in high-income countries in the same time period. [12]

Also, survival has been reported to be different between subsystems of health care. The three-year survival post-diagnosis has been reported to be 50% in the population without social security [13] against 64% at four years post-diagnosis in those with social security [14]. These results approach those seen in high-income countries at least 15 years ago. [4, 5]

In Mexico, poverty and inequity are important issues to resolve. The annual average household net-adjusted disposable income per capita is USD 13,891, while the OECD average is USD 30,563. The income of the richest individuals in the country (the top 20% of the population) is nearly 14 times as high as that of the poorest individuals in the country (the bottom 20%). [15]

Under the theoretical framework of the Social Determinants of inequalities in Health (SDH) [16], it is relevant that health and the success of a treatment will not only be a consequence of the medical treatment received, but also of a set of situations inside and outside the health services that facilitate or hinder recovery and maintenance of health.

Therefore, we are interested in analyzing the potential impact of these socioeconomic inequalities on the health of children, particularly in cancer survival probability.

### Context of the study

In Mexico, the main institution of social security is the Instituto Mexicano del Seguro Social (IMSS) with 58 million enrolled individuals [17]. Individuals are enrolled in the IMSS in either of two plans: the voluntary or the mandatory plan (for formal workers and their families, and students in high school or university). In 2015, the IMSS reported [18], 75% of the IMSS’s enrolled population earned less than USD 18 daily, 23% earned between USD 18 and 87, and only 2% earned more than USD 87 daily. (estimations based on [18]).

The IMSS has an infrastructure consisting of ~ 1500 primary-care units, 270 secondary hospitals, and 30 tertiary hospitals, which are organized into ten medical-service networks. [19]

This study analyzed the IMSS population living in Mexico’s central region, which includes 7 states and represents 27.1% of all children in Mexico. This region is served by two medical networks of services: the “La Raza” network serves the population of northern Mexico City and the states of Mexico and Hidalgo (8.1 million people), and the SXXI network serves the population of southern Mexico City and the states of Chiapas, Guerrero, Morelos, and Querétaro (6.03 million people). [20]

Due to geographical variations in the infrastructure of the services offered by the IMSS, there are potential differences in accessibility. To minimize the impact of these differences, the IMSS provides a series of social supports.

The two service networks analyzed, present important contrasts, such as geographic accessibility and health risk, which are relevant to the goals of this study. In the SXXI network, the distances from the residences of the population to the tertiary hospitals range from two km to > 1000 km (e.g., locations in Chiapas), whereas in the La Raza service network, the maximum distance is 200 km (estimations based on [19]). In terms of morbidity and mortality, according to Rodríguez-Abrego et al. [21] important differences in life expectancy were observed between the IMSS population covered by the SXXI network, which ranged between 73.1 years (Chiapas population) and 79.8 years (Quintana Roo population), with an IMSS national average of 76.4 years.

In this study, we analyzed socioeconomic inequalities in the survival of children (< 15 years old) with ALL insured by the IMSS, particularly focusing on the effect of the life path of children covered by social security and differences in the medical service networks of the IMSS.

## Methods

An analysis of five-year survival post-diagnosis was carried out to analyze socioeconomic inequalities for cohorts of minors (< 15 years old) with ALL diagnosed in 2007–2009 in two tertiary hospitals of the IMSS (La Raza and SXXI, serving the 7 states of the central region of Mexico); this analysis was performed by means of a Cox proportional risk model [22], adjusted also for clinical predictors of survival.

Acute lymphoblastic leukemia (ALL) diagnosis is classified as a high-risk or standard-risk according to clinical and laboratory indicators at the time of diagnosis and is reclassified according to the patient’s response to treatment. Characteristics for High risk diagnosis include the presence of one of these conditions: age < 1 year or > 10 years; hyperleukocytosis; immunophenotype of T cells; and any infiltration, mediastinal mass, or Down syndrome. For standard-risk diagnosis: absence of all the characteristics of high-risk diagnosis. [23]

The treatment is scheduled based on the risk of diagnosis, and in general, the therapy is less toxic and intensive for children with a standard-risk diagnosis than for children with a high-risk diagnosis. [23, 24]. The literature shows a treatment dropout rate in children with ALL of 24–64%, the dropout rate is higher in the initial stages of treatment and decreases in the advanced stages. In Mexican population of patients with ALL, the following dropout rates were reported: 30–40%. [25]

Therefore, the survival Cox model were adjusted by socioeconomic characteristics as well as these clinical characteristics of the minor: diagnosis risk of ALL, sex and age at diagnosis [23, 24], time lag [26] and year of diagnosis; urbanization and level of marginalization [27] of the community of residence; and the characteristics of the medical service network in which the child received care, such as: distance from tertiary hospital, type of secondary hospital, and distance from secondary hospital. [Estimations based on [19, 28].

### Material

The information was provided by the following:

1. The Registry of Children with Cancer (RCC) of the central region of the IMSS [29] provided incidence data for the cohorts of children diagnosed with ALL from 2007 to 2009; for each case, data on clinical, contact information, and the written, informed consent of the parents for the follow-up of the case. And, also the RCC provided data on survival conditions through December 31, 2014, based on data obtained from three sources: the clinical records in its tertiary hospitals, the national registry of mortality of those insured by the IMSS; and periodic telephone contact with family members of the children.
2. The Enrollment and fees Collection Directorate of the IMSS generated and provided the database of the insurance history of the parents through March 31, 2015.
3. The Division of Health Information of the IMSS provided the data on the hospital infrastructure of the IMSS through 2013.

### Analysis plan

For the estimation of the inequalities, two steps were followed [30]:

**Step 1. Identification of the variables of the socioeconomic stratification of the population.**

Under the theoretical framework of the Social Determinants of inequalities in Health (SDH) [16], the most important social stratifiers included in the SDH framework are: Level of income whose mechanism of differentiation between people is to generate differences in terms of access to material goods; Educational achievement that generates differences between people in terms of access to information and knowledge domain to benefit; the occupational status that generates differences related to paid work, prestige, power, privileges, and technical and social skills; the social class, understood as the ownership or control of productive resources and that reflects relations of subordination; gender: socially constructed characteristics of men and women that imposes rules of behavior; and race or ethnicity that generates differences when there are social groups that are discriminated against. [16]

In the case of Mexico formality of employment is a condition that allow access to social security (medical, social and services and economic benefits for individual and its family). Therefore, because social security is a changing condition built throughout the life of individuals, we include it as a determinant of inequalities in health.

In this study, based on the framework of SDH [16], three characteristics were included: income level, education level, and occupation of the parents. There was no information regarding an individual’s being a member of a group that was discriminated against.

To determine income level, two variables were analyzed: the availability of basic services in the home (potable water, indoor plumbing, and concrete or finished flooring) and monthly family income (USD dollars 2015) [31]. Parental education level was defined as the maximum level for either parent. Occupation included two characteristics: the maximum level of specialization of each parent [32] and the proportion of the children’s lives insured by the social security system. This proportion was determined by tracing the work history of the insured parent of each minor, from first entry into the IMSS system until March 31, 2015, for both the mandatory and the voluntary plans. Once the work history was traced, the proportion of the children’s life path was calculated.

All five socioeconomic variables were categorized because of the high correlations expected among them. The categories were determined according the meaning and the frequency of each variable. Schooling was categorized into 4, according to the completed grade: primary, high school, bachelor or technician career, university or higher; Occupation was categorized according to the International Standard Classification of Occupations (ISCO 2008) into 4 groups. The monthly family income was categorized by quartile of reported monetary income. The detail of the categories is presented in Table 1.

**Table 1:** Description of the variables of socioeconomic stratification and control

Variable	Operationalization
Characteristics of social stratification	
Maximum educational level of parents^a^	Highest educational level of parents. Ordinal variable: 1) university degree or higher; 2) bachelor’s degree or technical career; 3) some level of high school; and 4) some level of primary school or uneducated.
Monthly family income level^a^	Monthly family income reported in the Register of Childhood Cancers, converted into United States dollars (USD), on December 31, 2015, with an exchange rate of 20.6194 pesos per dollar [31]. Ordinal variable: grouped in quartiles of monthly income, 1) richest population to 4) poorest population.
Availability of basic services in the home^a^	Characteristics present in the domicile: potable water; indoor toilet (seat, cover, flushing system); and cement or finished floor. Dichotomous variable: 1) with all three characteristics; 0) absence of one of the characteristics.
Maximum occupational level (specialization of parents^a^)	Groupings based on the International Standard Classification of Occupations (ISCO) 2008 [32]. Ordinal variable: 1) Managers and professional occupations (includes ISCO group 1: managers and ISCO group 2: professionals); 2) Mid-level and support occupations: (includes ISCO group 3: Technicians and associate professionals and ISCO group 4: Clerical support workers); 3) Services, sales and skilled workers (includes ISCO group 5: Service and sales workers, ISCO group 6: Skilled agricultural, forestry and fishery workers, and ISCO group 7: Craft and related trades workers); 4) Machine operators; unskilled laborers and unemployed (includes ISCO group 8: Plant and machine operators and assemblers and ISCO group 9: Elementary occupations).
Proportion of minor’s life path that was IMSS-insured prior to ALL diagnosis ^b^	Proportion of days of minor’s life path (from date of birth to date of diagnosis of cancer) during which the minor was insured by social security. Ordinal variable: 1) 80–100%; 2) 50–80%; 3) 25–50%; and 4) < 25% of minor’s life path.
Control variables	
Clinical characteristics of minor	
Age group at ALL diagnosis^a^	Years between minor’s date of birth and date of diagnosis. Ordinal variable: 1) < 1; 2) 1 to < 5; 3) 5 to < 10; and 4) 10 to < 15 years old.
Sex^a^	Dichotomous variable: 1) male; 0) female.
ALL risk diagnosis ^a^	Presence of clinical characteristics at the time of diagnosis of acute lymphoblastic leukemia [23]. For high-risk diagnosis: age < 1 year or > 10 years; hyperleukocytosis; immunophenotype of T cells; and any infiltration, mediastinal mass, or Down syndrome. For standard-risk diagnosis: absence of all the characteristics of high-risk diagnosis. Dichotomous variable: 1) high risk diagnosis; 0) standard risk diagnosis.
Time lag in diagnosis^a^	Months between parents’ awareness of symptoms and date of diagnosis in a tertiary hospital [26]. Ordinal variable: 1) < 1 months; 2) 1 to 4 months; and 3) ≥4 months.
Diagnosis cohort^a^	Year in which diagnosis was confirmed by a specialist. Ordinary variable: 2007, 2008, 2009.
Proportion of treatment period of IMSS-insured minor (after ALL diagnosis) ^b^	Proportion of the treatment period of the minor (from date of cancer diagnosis to five-year follow-up) during which the minor was insured by social security (adjusted for date of death, when required). Ordinal variable: 1) 75–-100%; 2) 50 to < 75%; 3) 25 to < 50%; and 4) < 25%.
Characteristics of the community and medical service network	
Area of residence prior to ALL-diagnosis^a^	Area where patient resided prior to the date of diagnosis. Dichotomous variable: 1) Metropolitan (Mexico City and State of Mexico), 0) Provincial (states of Chiapas, Guerrero, Hidalgo, Morelos, and Querétaro).
Level of marginalization of the municipality of residence^a^	Marginalization, estimated by CONAPO [27] for the area of residence on the date of diagnosis, is based on the percentages of the following: population (≥15 years old) that is either illiterate or did not complete primary school; occupants in homes A) without indoor plumbing or toilet, B) without electric energy, C) without piped-in potable water, D) with some level of overcrowding, or E) with dirt floors; populations in locations with < 5000 inhabitants; and populations earning up to two minimal salaries. Ordinal variable: 1) very low or low; 2) medium; and 3) high or very high.
Network of services^a^	Medical service network corresponding to the location of family residence. Dichotomous variable: 1) SXXI service network covers the populations from the states of Chiapas, Guerrero, Morelos, Querétaro, and the southern part of Mexico City; 0) La Raza service network covers population of the states of Hidalgo, México, and the northern part of Mexico City.
Distance from tertiary hospital^c^	Distance (km) from the medical unit of primary care to the tertiary hospital that corresponds to the residence of patient. The distance was calculated by using Google maps [28] to determine the shortest route for a privately owned car. Ordinal variable): 1) < 20; 2) 20 to < 50; 3) 50 to < 200; and 4) ≥200 km.
Type of secondary hospital^d^	Type of resources available at the secondary hospital. The “Hospital General de Subzona” (HGS), has the four basic specialties plus an emergency department, 30–72 beds. The “Hospital General de Zona” (HGZ), has the same services as the HGS, as well as 72–144 beds and other specialties, such as trauma, ophthalmology, otorhinolaryngology, and subspecialties. The “Hospital General Regional” (HGR), provides medical attention to the population referred from the HGS and to some patients from the HGZ, provides the basic specialties and various subspecialties, and has > 200 beds. Ordinal variable: 1) HGR; 2) HGZ; and 3) HGS.
Distance from secondary hospital^c^	Distance (km) from primary-level medical unit to secondary hospital corresponding to location of patient’s residence. The distance was calculated by using Google maps [28] to determine the shortest travel distance for a privately owned car. Ordinal variable: 1) < 5; 2) 5 to < 10 km: 3) 10 to < 20 km; and 4) ≥20 km.

Sources of information:

^a^Register of Childhood Cancers, maintained by Clinical Epidemiology Research Unit-Pediatrics Hospital, contains clinical and socioeconomic data and contact information for minors and their families;

^b^Insurance history of the minor provided by the Enrollment and Fees Collection Division of the IMSS. (This information is not publicly available)

^c^Data on the latitude, longitude, and addresses taken from the directory of medical units of IMSS (2013);

^d^Database of Hospital Infrastructure in the IMSS (2013), provided by the Health Information Division of the IMSS. Available at: http://www.imss.gob.mx/directorio

**Step 2. Measuring the magnitude of inequality in five-year survival.**

We evaluated the slope of inequality of the probability of dying for the variables of social stratification that were relevant in a survival Cox model. The slope index of inequality shows the gradient of health across multiple subgroups [33], and is the coefficient of the linear regression of the relation between the level of health (in this case the probability of dying from ALL) and the hierarchical ranking of each socioeconomic category. [34]

We developed a Cox proportional risk model [22], based on the clinical predictors of ALL survival and the framework of social determinants of inequalities.

### Statistical analysis

The five-year survival post-diagnosis was calculated in natural days. For the patients who died within five years of diagnosis, the date of the last entry in the register corresponded to the date of death; for the cases lost to follow-up, the date of the last entry in the register was the last date registered in the clinical record of the tertiary hospital or the last date the patient was reported alive. For those who survived, the date was truncated at five years after diagnosis.

To estimate the probability of survival, tables and survival curves were generated using the Kaplan-Meier method. [22]

The model include clinical predictors such as age, diagnosis risk [23] and diagnosis time [26], community characteristics such as level of marginalization [27] and average distance from primary facilities to tertiary hospital [28], and the availability of infrastructure of the network of medical services [19]. (Table 1).

We focus on finding evidence of the presence of social inequalities and which determinants have the most influence, without forgetting that this determinant may be indirectly picking up the effect of another that was not significant. The mechanisms of influence of each determinant can be exclusive or overlap with that of another determinant, and if one were to interpret the individual effect separately, multiple causal patterns would have to be evaluated to avoid falling into bias. [35]

Therefore to identify potential modifying effects between determinants, we evaluate the variables that were statistically significant among health results, and attrition condition, and other variables were statistically significant (*p* < 0.05) in the descriptive analysis among the socioeconomic categories; and the inclusion of the corresponding interaction variable in stratified models was evaluated. [36]

To take care of the stability of the regression model and the potential problem of multicollinearity [36] among the variables of socioeconomic stratification was evaluated. We determined the precision of the coefficients and the stability of their magnitude, direction, and confidence interval by means of their inclusion in the regression model, both individually and when grouped.

The Cox PH model assumes that the hazard ratio for one individual is proportional to the hazard for any other individual, where the proportionality constant is independent of time [22]. For assessing the PH assumption we followed three approaches: 1. The graphical approach: comparing observed and predicted survivor curves, observed curves are derived for categories of the variable being assessed, without the inclusion of this variable in the model; the predicted curves are derived with this variable included in the model. If both curves are similar, then the PH assumption is reasonable; 2. The goodness-of-fit tests for each variable in the model, adjusted for the other variables in the model, a nonsignificant *p*-value suggests that the PH assumption is reasonable [22]; and 3. The Schoenfeld residue test for each variable; evaluating that the residuals for each covariate will not be related to survival time. [22]

The difference between the survival curves was evaluated by the logarithm of ranges (the null hypothesis is a common survival curve) and Wilcoxon tests (where early failures receive more weight than later failures). [22]

Finally, we conducted a scenario sensitivity analysis [22] to evaluate the robustness of the results. First, we tested the randomness of the loss to follow-up [37] and identified associated variables by means of the same Cox regression model using the loss to follow-up as the dependent variable. With each variable resulting in statistical significance, we defined scenarios, according to better socioeconomic characteristics, i.e., the status set to alive, and the worst socioeconomic position, i.e., the status set to death. Then, we repeated the Cox regression model and probed the stability of the results: the statistical significance and the direction and magnitude of the coefficients.

For the statistical analysis, the program Stata v. 11 (Stata Corp LP, USA) was used.

## Results

### Description of the study population

We analyzed the information of 294 children diagnosed with ALL in the successive cohorts of 2007–2009.

Table 2 shows the comparative distribution of the characteristics according to life status at five years post-diagnosis and the univariate hazard ratio of dying for each category.

**Table 2:** Clinical and socioeconomic characteristics of the study population

Variables and categories	Total (*n*)	% of Total	Survival (median, in years)	Status at five-year follow-up				Hazard ratio (CI_95%_)at five years*
				Dead	Censored cases			
					Alive	Attrition	*p*	
Children’s life status	294	100%	4.1	43.9%	44.6%	11.6%		
Characteristics of social stratification of the home								
Maximum educational level (parents)								
University degree or higher	63	21.4%	3.7	46.0%	42.9%	11.1%	0.355	(Reference group)
Bachelor’s/technical degree	109	37.1%	4.4	45.0%	47.7%	7.3%		0.96 (0.60, 1.51)
High school	101	34.4%	3.8	38.6%	44.6%	16.8%		0.86 (0.53, 1.39)
Primary school or less	21	7.1%	4.4	57.1%	33.3%	9.5%		1.21 (0.62, 2.38)
Monthly family income level, median [range] USD ^a^								
Q4: 644 [481, 2910]	72	24.5%	4.9	31.9%	50.0%	18.1%	0.125	(Reference group)
Q3: 355 [294, 477]	67	22.8%	3.3	53.7%	40.3%	6.0%		1.82 (1.08, 3.08)*
Q2: 218 [165, 291]	80	27.2%	3.9	48.8%	41.3%	10.0%		1.61 (0.96, 2.70)
Q1: 126 [73, 162]	75	25.5%	3.8	41.3%	46.7%	12.0%		1.44 (0.84, 2.47)
Availability of basic services in the home^b^								
All three services	269	91.5%	4.3	42.0%	46.8%	11.2%	0.035	(Reference group)
Without at least one	25	8.5%	2.1	64.0%	20.0%	16.0%		1.88 (1.12, 3.18)*
Maximum occupational level (parents)								
Managers and professionals	29	9.9%	4.9	37.9%	51.7%	10.3%	0.473	(Reference group)
Mid-level and support workers	128	43.5%	3.6	49.2%	41.4%	9.4%		1.43 (0.76, 2.72)
Service, sales, and skilled workers	68	23.1%	3.4	45.9%	39.7%	14.7%		1.40 (0.71, 2.79)
Machine operators; unskilled laborers and unemployed	69	23.5%	4.9	34.8%	52.2%	13.0%		0.92 (0.45, 1.88)
Proportion of minor’s life insured by IMSS prior to ALL diagnosis								
80–100%	149	50.7%	5.0	36.2%	55.7%	8.1%	0.003	(Reference group)
50–80%	64	21.8%	3.5	42.2%	40.6%	17.2%		1.27 (0.80, 2.02)
25–50%	34	11.6%	2.5	61.8%	26.5%	11.8%		2.02 (1.22, 3.36)*
0–25%	47	16.0%	2.3	57.4%	27.7%	14.9%		2.09 (1.32, 3.32)*
Clinical characteristics of minor								
Age group at ALL diagnosis (years)								
< 1	9	3.1%	1.1	77.8%	22.2%	0.0%	0.000	4.00 (1.80, 8.91)*
1 to < 5	126	42.9%	5.0	34.9%	57.1%	7.9%		(Reference group)
5 to < 10	81	27.6%	4.7	44.4%	46.9%	8.6%		1.33 (0.86, 2.07)
10 to < 15	78	26.5%	2.5	53.8%	24.4%	21.8%		1.92 (1.26, 2.94)*
Sex								
Female	132	44.9%	3.9	42.4%	47.0%	10.6%	0.734	(Reference group)
Male	162	55.1%	4.0	45.1%	42.6%	12.3%		1.04 (0.74, 1.48)
ALL risk diagnosis								
Standard risk	143	48.6%	4.9	35.0%	56.6%	8.4%	0.000	(Reference group)
High risk	151	51.4%	2.5	52.3%	33.1%	14.6%		1.87 (1.31, 2.67)*
Time lag in diagnosis								
< 1 month	125	42.5%	3.8	44.8%	44.0%	11.2%	0.465	(Reference group)
1 to 4 months	139	47.3%	3.8	45.3%	41.7%	12.9%		1.07 (0.74, 1.54)
> 4 months	30	10.2%	5.0	33.3%	60.0%	6.7%		0.67 (0.35, 1.33)
Diagnosis cohort								
2007	103	35.0%	2.8	46.6%	34.0%	19.4%	0.006	(Reference group)
2008	94	32.0%	4.9	38.3%	55.3%	6.4%		0.67 (0.44, 1.04)
2009	97	33.0%	4.3	46.4%	45.4%	8.2%		0.85 (0.57, 1.28)
Proportion of treatment period of IMSS-insured minor (after ALL diagnosis)								
75–100%	258	87.8%	3.8	47.3%	44.6%	8.1%	0.000	(Reference group)
50% to < 75%	17	5.8%	4.6	23.5%	47.1%	29.4%		1.505 (0.14, 16.6)
25 to < 50%	11	3.7%	2.9	18.2%	36.4%	45.5%		1.687 (0.19, 15.1)
< 25%	8	2.7%	4.8	12.5%	50.0%	37.5%		4.112 (0.57, 29.4)
Characteristics of the community and the medical service network								
Area of residence								
Metropolitan	252	85.7%	4.3	43.3%	46.0%	10.7%	0.347	(Reference group)
Provincial	42	14.3%	2.7	47.6%	35.7%	16.7%		1.31 (0.81, 2.11)
Level of marginalization of the municipality of residence								
Very low or low	243	82.7%	4.3	43.2%	45.7%	11.1%	0.589	(Reference group)
Medium	42	14.3%	3.3	45.2%	38.1%	16.7%		1.22 (0.75, 1.99)
High or very high	9	3.1%	3.6	55.6%	44.4%	0.0%		1.42 (0.58, 3.49)
Network of services								
La Raza	205	69.7%	4.6	41.0%	48.3%	10.7%	0.148	(Reference group)
SXXI	89	30.3%	2.6	50.6%	36.0%	13.5%		1.45 (1.01, 2.09)*
Distance from tertiary hospital (km)								
< 20	119	40.5%	3.9	47.1%	43.7%	9.2%	0.037	(Reference group)
20 to < 50	96	32.7%	4.4	40.6%	46.9%	12.5%		0.85 (0.56, 1.28)
50 to < 200	58	19.7%	4.9	43.1%	50.0%	6.9%		0.91 (0.57, 1.46)
≥ 200	21	7.1%	1.1	42.9%	23.8%	33.3%		1.32 (0.65, 2.67)
Type of secondary-level hospital^c^								
General; regional	83	28.2%	4.6	45.8%	44.6%	9.6%	0.658	(Reference group)
General; zone	205	69.7%	4.1	42.4%	45.4%	12.2%		0.90 (0.62, 1.32)
General; subzone	6	2.0%	0.9	66.7%	16.7%	16.7%		2.24 (0.80, 6.29)
Distance from secondary hospital (km)								
< 5	176	59.9%	3.9	44.3%	44.9%	10.8%	0.568	(Reference group)
5 to < 10	56	19.0%	3.2	42.9%	50.0%	7.1%		1.19 (0.77, 1.83)
10 to < 20	44	15.0%	4.3	45.5%	36.4%	18.2%		0.78 (0.46, 1.34)
≥ 20	18	6.1%	4.4	50.0%	33.3%	16.7%		0.85 (0.37, 1.94)

^a^Exchange rate at December 31, 2015: 20.6194 pesos per US dollar. Available at http://www.banxico.org.mx/portal-mercado-cambiario/

^b^Potable water, indoor plumbing, and concrete or finished flooring

^c^The Hospital General de Subzona (HGS) has the four basic specialties plus an emergency unit and 30–72 beds. The Hospital General de Zona (HGZ) provides the same services as the HGS, as well as other specialties, such as trauma, ophthalmology, otorhinolaryngology, and subspecialties. The Hospital General Regional (HGR) provides medical attention to the population derived from the HGS and to some patients from the HGZ and provides basic specialties and various subspecialties

*Statistically significant (p ≤ 0.05)

Nearly 60% of the parents of the 294 children in the study had at least a bachelor’s education, 34.4% had higher education and only 7.1% had a primary-level education or lower.

The median of the reported monthly family income(s) was USD 291; the median income of families in the 4th quartile was 5.1-fold greater than that of families in the 1st quartile, with 90% of families reporting a monthly income of < USD 679.

When the income distribution of the study population was compared with that of the entire eligible population of the center region [18], it was observed that the median income was similar, but the proportions with incomes in different categories were as follows: income of less than USD 2100: 0.5% according to the IMSS data vs. 8.8% of the study population); incomes greater than USD 15,000: 23.6% according to the IMSS data vs. 8.5% of the study population; and salaries over USD 30,000: 7.5% according to the IMSS data vs. less than 1% of the study population). Thus, the distribution was truncated for incomes greater than 15 thousand pesos and was overrepresented for low incomes.

Nearly 91.5% of the families had potable water, adequate indoor toilet facilities, and either concrete or finished flooring; the remaining 8.5% lacked some services, most frequently potable water within the home (15 cases). The most frequently occurring occupational level consisted of mid-level and support activities (43.5%), and the least frequently occurring consisted of managers and professional occupations (~ 10% of the families).

The database on the insurance history of the parents included 18,000 entries of changes in employment status. Prior to diagnosis, half of the children had been covered by social security for > 80% of their lives prior to their ALL diagnosis: 21.8% of the children for more than 50% of their lives and 11.6% for at least 25% of their lives. However, 16% were covered for less than 25% of their lives.

During the treatment period, 87.8% of the families continued to be insured for at least 75% of the period of the patient’s treatment.

A total of 51.4% of the children had a high-risk diagnosis of ALL: 38.7% due to the age group, 30.7% due to having a different risk factor (the most frequent being hyperleukosynthesis, 14%), 24% due to having two risk factors, and 6.6% due to having up to 4 risk factors (data not shown). Nearly 90% of cases were diagnosed before 4 months since awareness of symptoms.

A total of 85.7% of the study population resided in the metropolitan area (Mexico City 32.7% and State of Mexico 53.4%) and 14.3% in other states. Only 3.1% of the study population resided in municipalities of medium marginalization, and none had high or very high marginalization.

In total, 69.7% of the cases were received in the La Raza service network and 30.3% in the SXXI network.

In 7.1% of families, the distance to the tertiary hospital from their habitual residence was greater than 200 km. The median distance of a residence from a tertiary hospital was 21.9 km (range: 1.1 km, 1090 km). The median distance was significantly different between the networks of services (18.2 km for SXXI vs. 27.2 km for La Raza, *p* = 0.017); 23.1% of families covered by the SXXI network lived at > 200 km distance, whereas none of families covered by the La Raza network did. For 65% of the families, the estimated travel time by private automobile was < 1 h; for 27%, the travel time was up to 2 h; and for the remaining 8%, it was > 2 h (data not shown).

The median distance to the secondary hospital was 4 km (range: 0 km to 105 km), for 6.1% of the study population the distance was more than 20 km. the estimated travel time to secondary hospital was less than 30 min for 87% of patients, less than 1 h for 12%, and more than 1 h for 0.3% (data not shown). According to the type of secondary hospital, 99% had emergency services, pharmacy, laboratory and X-rays; in 96% of the cases, there was a pediatrician; and in 57% of the cases, there were intensive care services.

### Survival results

It was found that 44.6% of the children had survived at least five years post-diagnosis, 43.9% had died, and 11.6% were lost to follow-up. The median survival was 4.1 years; the lowest median survival (< 2 years) was observed in patients with access to a subzone secondary hospital (0.9 years), and 1.1 years: in patients < 1 year old and in patients living > 200 km from a tertiary hospital.

The median survival of those with a standard-risk diagnosis of ALL was 2-fold greater than that for those with a high-risk diagnosis (4.9 vs. 2.5 years). The median survival was 2-fold greater for patients living with all basic services than for those living without at least one (4.3 vs. 2.1 years) and for those insured > 80% of their lives than for those insured < 25% of their lives.

Of those who had died, their deaths had been confirmed through institutional mortality records (92.7%), by telephone with a family contact (4.4%), or by a note in the medical record (2.9%). Of the identified deaths, 83% had been registered in tertiary hospitals, 8.5% in secondary hospitals, and 1.5% in primary care units; 7% died in non-IMSS facilities.

It was observed that 100% of the deaths in the population of quartile 4 were registered in tertiary units, against 73, 94 and 91% of the deaths in the populations in quartiles 1 to 3, respectively (data not shown).

### Variables considered with a potential modifier effect

Variables with a proportion of losses greater than 20% and differences statistically significant was observed in the 2007 cohort, in children over 10 years of age, in provincial residents, and in children whose homes were greater than 200 km away from a tertiary-hospital. A greater proportion of deaths occurred among those who had a high-risk diagnosis of ALL and who lacked basic services in the home. There were a proportion of survivors greater than 50% and among cases with a pre-diagnostic insurance trajectory for more than 50% of their lives and in those with a post-diagnosis insurance greater than 25% of the treatment time. Additionally, other variables with a significant risk ratio were SXXI network, age less than 1 year or greater than 10 years, and income in quintile 3.

The follow-up time in cases censored before 5 years between service networks was significantly higher only in high-risk diagnosis of ALL patients treated in the SXXI network, and the distance from the usual residence to the tertiary hospital was statistically higher in the patients treated in the SXXI network, particularly in the high-risk diagnosis of ALL patients censored for loss.

Therefore, we evaluated 9 variables as a potential modifiers: family income level, availability of basic services at home, proportion of children’s life IMSS insured before and after ALL diagnosis, ALL risk diagnosis, diagnosis cohort, network of services, and distance to tertiary hospital. These variables were evaluated in the Cox model.

We only found a potentially modifying effect in two variables, the ALL diagnosis risk with the type of hospital and with the network of services. However, only the interaction variable created with the risk ALL diagnosis and the network of medical services was significant, even after adjusting for the distance from the unit and the resources of the secondary hospital. This result could represent differences in the clinical management of patients with High-risk diagnosis of ALL between the two tertiary hospitals (SXXI and La Raza).

### Final model

The adjusted Cox regression model is presented in Table 3.

**Table 3:** Adjusted Cox regression model^a^ for the survival of children with acute lymphoblastic leukemia (2007–2009 cohorts)

Variable	Category	Hazard ratio (CI_95%_)
Characteristics of social stratification of the home		
Maximum educational level (parents)	University degree or higher	(Reference group)
	Bachelor’s/technical degree	0.6 (0.37, 1.13)
	High school	0.7 (0.35, 1.22)
	Primary school or less	1.0 (0.41, 2.28)
Monthly family income level	4th quartile	(Reference group)
	3rd quartile	1.8 (0.99, 3.09)
	2nd quartile	1.6 (0.90, 2.95)
	1st quartile	1.1 (0.59, 2.21)
Availability of basic services in the home	All three present	(Reference group)
	At least one not present	1.9 (1.00, 3.75)*
Maximum occupational level (parents)	Managers and professionals	(Reference group)
	Mid-level and support workers	1.4 (0.65, 2.89)
	Service, sales and skilled workers	1.3 (0.55, 3.04)
	Machine operators; unskilled laborers and unemployed	0.8 (0.34, 2.09)
Proportion of minor’s life insured by IMSS prior to ALL diagnosis	80–100%	(Reference group)
	50 to < 80%	1.3 (0.74, 2.41)
	25 to < 50%	2.2 (1.18, 4.28)*
	< 25%	2.4 (1.35, 4.42)*
Clinical characteristics of minor		
Age at ALL diagnosis (years)	< 1	3.1 (1.21, 7.81)*
	1 to < 5	(Reference group)
	5 to < 10	0.9 (0.55, 1.51)
	10 to < 15	0.9 (0.48, 1.70)
Sex	Female	(Reference group)
	Male	1.0 (0.69, 1.52)
ALL risk diagnosis	Standard risk	(Reference group)
	High risk	1.3 (0.81, 2.20)
Diagnosis cohort	2007	(Reference group)
	2008	0.7 (0.45, 1.19)
	2009	1.0 (0.63, 1.56)
Characteristics of the community and medical service network		
Network of services	La Raza network	(Reference group)
	SXXI network	0.7 (0.31, 1.36)
Interaction: diagnosis risk and medical service network	No high risk in SXXI network	(Reference group)
	High risk in SXXI network	2.6 (1.12, 5.95)*
Distance from tertiary hospital (km)	< 20	(Reference group)
	20 to < 50	1.0 (0.65, 1.60)
	50 to < 200	0.9 (0.52, 1.70)
	≥200	0.7 (0.25, 1.73)
Type of secondary hospital	Hospital General Regional	(Reference group)
	Hospital General de Zona	0.9 (0.59, 1.52)
	Hospital General de Subzona	2.0 (0.55, 7.15)

^a^Statistics of the model: observations, 294; deaths, 129; likelihood test (log): -663.4

*Statistically significant; *p* = 0.00005

Two socioeconomic variables were statistically relevant: the risk of dying was 1.9-fold higher for patients lacking basic services in the home, and children who had been IMSS-insured for less than half their lives had more than double the risk of dying than those who had been insured for their entire lives.

The relevant clinical variables were that the < 1-year-old age group had a risk of dying, which was 3.1-fold higher than the risk for minors 1–5 years old.

For the variables related to community and the network of medical services, the risk of dying was 2.6-fold greater for those receiving care in the SXXI network, but only for those who had high-risk diagnoses for ALL.

### Model evaluation

The evaluation of collinearity showed that neither the direction nor the significance of the coefficients was altered; in general, the effect was a tightening of the CI. Therefore, the five socioeconomic variables were retained in the final model.

The graphic analysis of the residuals and the verification of the fulfillment of the assumptions of proportionality of the model were analyzed; the overall test was not statistically significant (0.571), meaning that the variables included in the model were independent of time, and thus satisfying the assumptions of the Cox model.

**Fig. 1.**
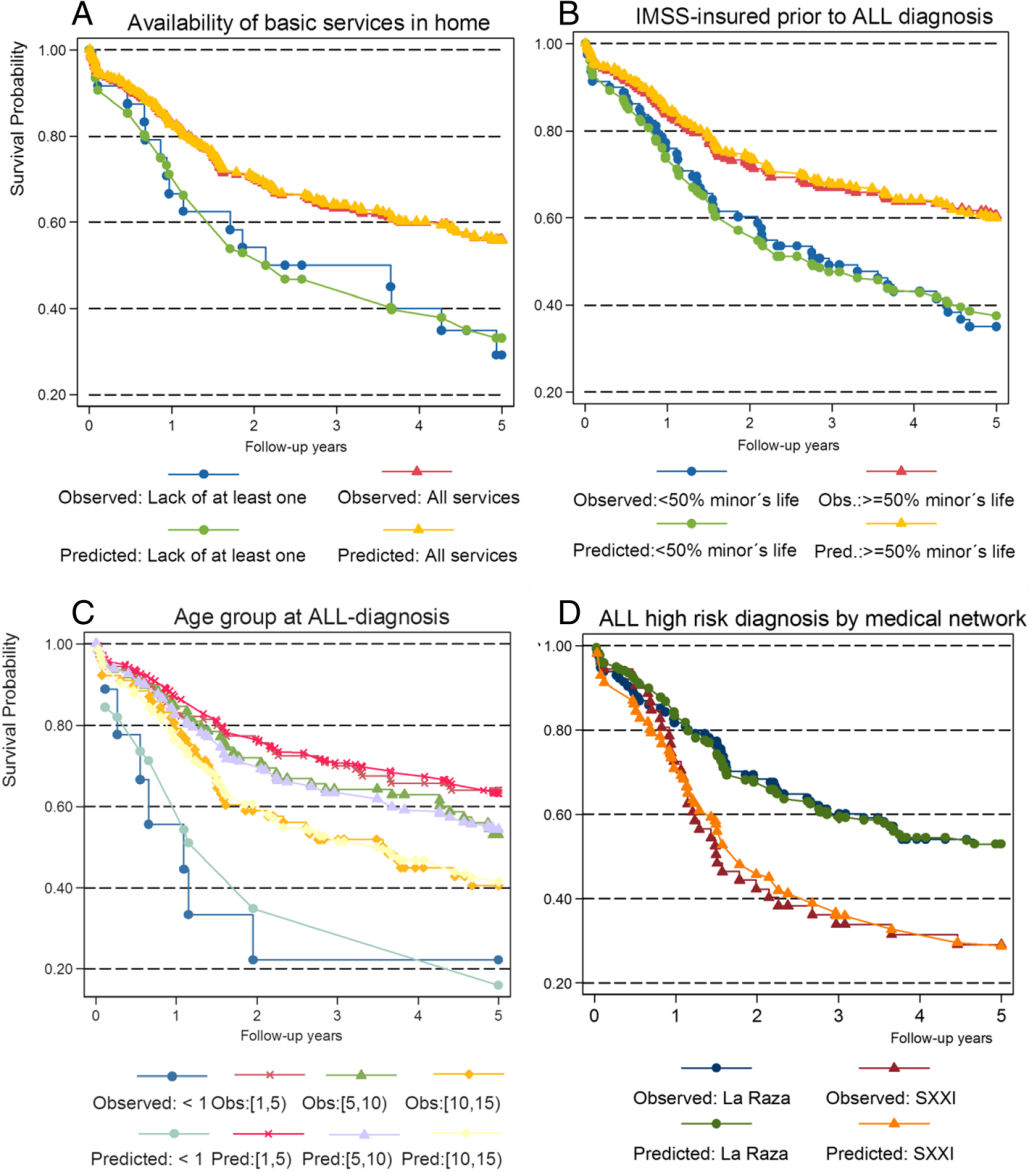
Survival curves for significant socioeconomic and clinical variables

Figure 1 shows the comparison between the unadjusted Kaplan-Meier survival curves and the Cox model of risk for the variables that were statistically significant: Availability of basic services at home (Fig. 1, Panel A), Proportion of children’s life IMSS-insured (Fig. 1, Panel B); Age groups (Fig. 1, Panel C), and ALL risk diagnosis by network services (Fig. 1, Panel D)). We observe that the PH assumptions are reasonable, since for each category, the expected and observed curves are similar.

Log rank test resulted statistically significant for the four variables show in Fig. 1, in Panel A, it is appreciated that Patients with a lack of basic services in the home begin with a 20% lower chance of living, and this chance decreases up to 50%. Panel B shows that those children who had been insured for < 50% of their lives had lower chance of surviving; Panel C shows that children under 1 have a different survival trajectory than the other children, and Panel D shows that children with a high-risk diagnosis attended in the SXXI service network start with a 10% lower chance of survival, which reaches a 30% lower chance of survival after the second year.

In the sensitivity analysis including only the cases with complete follow-up, we found that the results held robust. The test of the randomness of the loss to follow-up resulted in significance for three variables: family income level, distance from tertiary hospital and diagnosis cohort. We probed scenarios with these variables, comparing the results with those of the original model. We found that the results maintained for the variable -proportion of children’s life IMSS insured, but not for the interaction variable between the network of services and ALL diagnosis risk, neither for the variable availability for basic services at home. The variable family income resulted nearly with statistically significant, however only for two categories and the coefficient and CI resulted similar.

Regarding the sensitivity analysis we considered our results are robust for the variable proportion of children’s life IMSS insured, and for the other variables, the results should be taken more conservatively.

In additional file 1 we show the details of the sensitivity analysis.

**Fig. 2:**
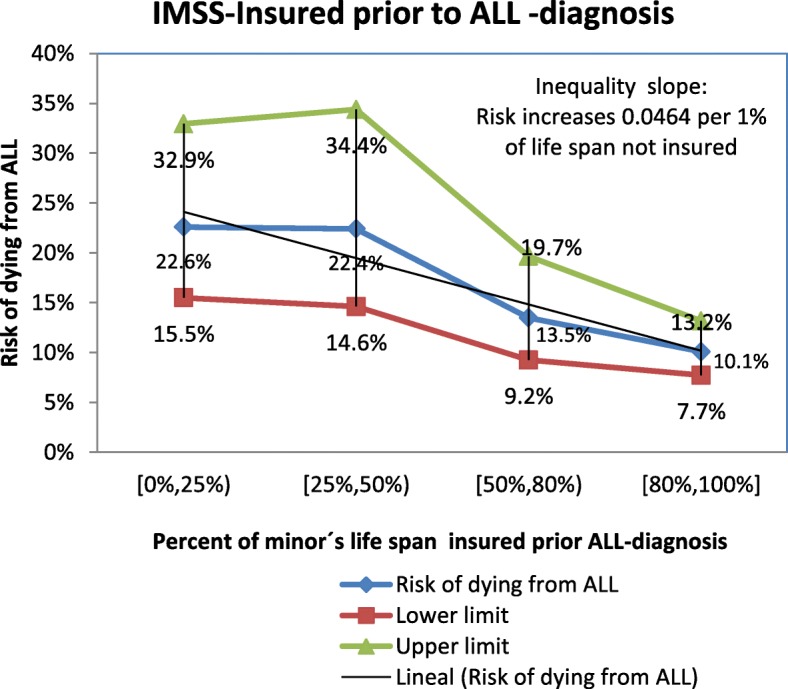
Inequality slope for the risk of dying for children with ALL

The slope of inequality in the risk of dying from ALL was estimated the variable that resulted relevant in the final Cox model and robust in the sensitivity analysis. For each level of the proportion of children’s life IMSS- insured, a slope of 4.64% was estimated. In terms of probabilities, Fig. 2 shows that children who had been insured for < 25% of their lives had a 2.2-fold greater probability of dying than those who had been covered for at least 80% of their lives (22.6% vs. 10.1%).

## Discussion

The results of this study did not show inequalities in the survival of children with ALL related to family income or the parents’ occupational or educational levels. However, an important socioeconomic factor that influenced survival was the proportion of the children’s lives covered by the social security system prior to their ALL diagnosis. To explain the effect of social security on the probability of the survival of the child, multiple mechanisms should be analyzed. [35]

We hypothesize two mechanisms: one directly related to children’s health and another indirectly related to the conditions of parents’ employment. The direct mechanism is the effect of health care throughout the life path of the child. Children insured for a shorter period of their lives would likely have less access to health care (preventive and curative services), have counted the mother of the child with permission for breastfeeding; and other social services, such as child daycare centers. In particular, insurance coverage prevents family resources that are required to cover basic necessities from being diverted for health care, which could signify economic and social consequences for the family. [38]

The indirect mechanism, related to employment conditions refers that formal employee-employer relationship [39, 40] ensures coverage by the social security system; this arrangement confers economic stability to the family by providing medical, economic, and social benefits, such as promoting compliance with labor norms. Prior studies have described how, during a child’s early years, the social circumstances of the parents at the level of social support structures for health care affect the early cognitive, emotional, and social development of children. [41]

Both mechanisms result equally relevant for policy considerations.

Contrary to the initial hypothesis and the findings of studies such as those of Son [8] and Lightfoot [9], no convincing evidence of inequalities being associated with the characteristics of income level, educational level, or parental occupation was found in the current study.

We hypothesized that this lack of evidence may be due to the loss to follow-up for the richest families (4th quartile) and may be due to survival; therefore, the gaps in the survival between income extremes could be underestimated.

We supposed this hypothesis, because, as we explained before, we think the income reported is overrepresented for low incomes comparing the total population covered by the IMSS in the central region of Mexico. In this study, we only include monetary income, we do not include other sources of income. In Mexico, it is reported that 80% of household income comes from monetary income, 13% from the value of assets and the rest from self-consumption and remuneration in kind. [42], this proportion is greater for the two poorest deciles. We use as a proxy variable of non-monetary income, the variable the availability of basic services in the home, mainly to catch the absence of this non-monetary income in the poorest households. We found that 8.5% of our population did not have basic services in the home and that the difference in survival was significant. So we think that the variable turned out to be a good proxy of non-monetary income.

Also we observed that all registered deaths in the 4th income quartile occurred in the tertiary hospitals of the IMSS vs. 91, 94, and 73% for the 3rd, 2nd, and 1st quartiles, respectively. From this result, one may suppose that attrition in the 4th quartile may have been due to survivors; therefore, survival in the 4th quartile (the richest) may have been even higher.

However, we probed this hypothesis in the sensitivity analysis, and the results were not robust. Therefore, the finding of an absence of significant gaps between family income strata in the population insured by the IMSS needs to be more thoroughly investigated.

### Limitations of the study

There are two important limitations related to information sources in this study. The most important limitation is that evidence was found that the loss of cases to follow-up is biased towards underreporting the survival of the highest strata of the study population, whereby the results indicating an absence of social inequalities in survival could be biased. However, we conducted a sensitivity analysis, and the results were not definitive.

The second limitation is related to the lack of data on the membership of social groups, particularly indigenous ethnic groups, who face cultural barriers to accessing care for minors.

The frequency of such groups is estimated to be important in the states of Guerrero and Chiapas (SXXI network).

### Future studies

The overall survival rate at five years for children with ALL in this study (53.7%) was much lower than that for children with ALL in high-income countries (90%), only reaching the results obtained for these countries in 2000 [4–6], a finding that merits further study.

Due to the evidence of potential differences between the service networks in the results for patients with high-risk diagnoses, future studies are needed to explore the following: the implementation of and compliance with the distinct treatment protocols, on the part of both the medical staff and the families; the adherence to treatment instructions on part of the families; and the utilization of health and auxiliary services. Similarly, given the aggressiveness of the infirmity and treatments, it will be important for subsequent studies to include indicators of quality of life for patients during treatment and for survivors.

## Conclusion

Regarding the survival of children with ALL receiving care in the subsystem of social security in Mexico, we did not identify inequalities according to expected socioeconomic factors (family income and parental educational and occupational level). However, an important protective trend was observed with respect to the proportion of children’s lives covered by social security before their cancer diagnosis. This result could be evidence of the role of social security as a mechanism of wealth redistribution and social mobility.

We conceptualized social security not as a static and dichotomic condition but rather as a dynamic and continuous characteristic, constructed along the lives of families and individuals. Therefore, results could be extrapolated for all Mexican populations, including those who have never had social security.

### Policy recommendations

We found a protective effect of social security, adding evidence of its effectiveness as a redistributing mechanism of wealth and promoter of social mobility. To extend the social security benefits to all Mexican population could be reflect in better health outcomes at medium and long term.

In the long term, the cost of implementing a social security system with universal coverage would be offset by the gains in health and the economics of the illnesses and deaths prevented.

In the medium term, the gaps and legal contradictions in the health care of minors that are associated with the employment status of parents must be reviewed, because represents a greater cost for the attention of the complications generated by the interruption of the treatment, and a relevant social cost during the search for care options for minors, which further aggravates the condition of unemployment or a lack of social protection for the family.

At the national level, it is necessary to maintain a routine, systematized, and high-quality register of the results of the health of the children with cancer to evaluate both the effectiveness of the medical attention given in such cases and the necessity of improving access to health care.

## Additional file

Additional file 1: Sensitivity analysis. Results of analysis of sensitivity detailed. (DOCX 87 kb)

## Abbreviations

ALL: Acute lymphoblastic leukemia
CONAPO: Consejo Nacional de Población (National Population Council)
IMSS: Instituto Mexicano del Seguro Social (Mexican Institute of Social Security)
ISCO: International Standard Classification of Occupations
La Raza: Medical service network of the National Medical Center La Raza
SXXI: Medical service network of the National Medical Center Siglo XXI
