# Correction to: socioeconomic inequalities in survival of children with acute lymphoblastic leukemia insured by social security in Mexico: a study of the 2007-2009 cohorts

**DOI:** 10.1186/s12939-019-0956-8

**Published:** 2019-04-08

**Authors:** Angélica Castro-Ríos, Hortensia Reyes-Morales, Blanca E. Pelcastre-Villafuerte, Mario E. Rendón-Macías, Arturo Fajardo-Gutiérrez

**Affiliations:** 1 Unidad de Investigación en Epidemiología Clínica, Hospital de Pediatría, Centro Médico Nacional SXXI, Instituto Mexicano del Seguro Social, Avenida Cuauhtémoc 330, Col. Doctores, Ciudad de México, Mexico; 2 Centro de Investigación en Sistemas de Salud, Instituto Nacional de Salud Pública, Cuernavaca, Morelos Mexico; 3 Public Health Department, Universidad Panamericana, Ciudad de México, Mexico

**Correction to: International Journal for Equity in Health**

**https://doi.org/10.1186/s12939-019-0940-3**

Following publication of the original article [1], the author reported her name has been erroneously spelled as Blanca E. Pelcastre. The full name is Blanca E. Pelcastre-Villafuerte.

It has been corrected in the original article as well.

The publisher apologizes for any inconvenience caused by this error.
