# Supplementary material for: Socioeconomic inequalities in survival of children with acute lymphoblastic leukemia insured by social security in Mexico: a study of the 2007–2009 cohorts
Source: Int J Equity Health. 2019 Mar 4;18:40. doi: 10.1186/s12939-019-0940-3 (PMC6399870; doi:10.1186/s12939-019-0940-3)
Supplement: Supplementary file 1 — Sensitivity analysis. Results of analysis of sensitivity detailed. (DOCX 87 kb) [file 12939_2019_940_MOESM1_ESM.docx]

**Additional file 1.**

**SENSITIVITY ANALYSIS**

The robustness of the results was evaluated through a sensitivity analysis. First, the consistency of the results was evaluated if only the cases with complete follow-up were included [Table 1].

It is observed that two variables lost statistical significance: the interaction between ALL risk diagnosis & the network of medical services; and the Availability of basic services in home. While the results of age and the proportion of children´s life IMSS-insured prior ALL diagnosis held its direction and magnitude.

[Table 1]

**Table 1. Sensitivity analysis comparing original survival Cox model against model using only cases with complete follow-up**

| **Variables** | **A. Original survival Cox model** | | | | **B. Survival Cox model using only cases with complete follow-up** | | | | |
| --- | --- | --- | --- | --- | --- | --- | --- | --- | --- |
|  | **Failure=Death** | | | | **Failure=Death** | | | | |
|  | Observations: 294, Failures: 129, P=0.000 | | | | Observations: 260, Failures: 129, P=0.000 | | | | |
|  | **Hazard ratio** | **CI 95%** | | | **Hazard ratio** | **CI 95%** | | | **Changes**  **vs A** |
| **Sex** |  |  |  |  |  |  |  |  |  |
| Female |  |  |  |  |  |  |  |  |  |
| Male | 1.0 | 0.69 | 1.52 |  | 1.2 | 0.81 | 1.85 |  |  |
| **Age at ALL-diagnosis (years)** |  |  |  |  |  |  |  |  |  |
| <1 | 3.1 | 1.21 | 7.81 | * | 2.8 | 1.11 | 7.03 | * | No change |
| 1 to < 5 |  |  |  |  |  |  |  |  |  |
| 5 to < 10 | 0.9 | 0.55 | 1.51 |  | 0.9 | 0.54 | 1.49 |  |  |
| 10 to <15 | 0.9 | 0.48 | 1.70 |  | 1.0 | 0.50 | 1.92 |  |  |
| **Network of services** |  |  |  |  |  |  |  |  |  |
| La Raza network |  |  |  |  |  |  |  |  |  |
| SXXI network | 0.7 | 0.31 | 1.36 |  | 0.6 | 0.31 | 1.35 |  |  |
| **ALL risk diagnosis** |  |  |  |  |  |  |  |  |  |
| Standard-risk |  |  |  |  |  |  |  |  |  |
| High-risk | 1.3 | 0.81 | 2.20 |  | 1.5 | 0.91 | 2.55 |  |  |
| **Interaction: ALL risk & network** |  |  |  |  |  |  |  |  |  |
| No high-risk dx in SXXI network |  |  |  |  |  |  |  |  |  |
| High-risk dx in SXXI network | 2.6 | 1.12 | 5.95 | * | 2.3 | 0.99 | 5.31 |  | Lost significance |
| **Monthly family income level** |  |  |  |  |  |  |  |  |  |
| 4th quartile |  |  |  |  |  |  |  |  |  |
| 3rd quartile | 1.8 | 1.00 | 3.09 |  | 1.3 | 0.72 | 2.31 |  |  |
| 2nd quartile | 1.6 | 0.90 | 2.95 |  | 1.2 | 0.63 | 2.20 |  |  |
| 1st quartile | 1.1 | 0.59 | 2.21 |  | 0.8 | 0.39 | 1.60 |  |  |
| **Availability of basic services in home** |  |  |  |  |  |  |  |  |  |
| All three present |  |  |  |  |  |  |  |  |  |
| At least one not present | 1.9 | 1.00 | 3.75 | * | 0.5 | 0.26 | 0.99 |  | Lost significance |
| **Maximum educational level (parents)** |  |  |  |  |  |  |  |  |  |
| University degree or higher |  |  |  |  |  |  |  |  |  |
| Bachelor school/ technical | 0.6 | 0.37 | 1.13 |  | 0.6 | 0.35 | 1.08 |  |  |
| High school | 0.7 | 0.35 | 1.22 |  | 0.7 | 0.37 | 1.35 |  |  |
| Primary school or less | 1.0 | 0.41 | 2.28 |  | 1.0 | 0.40 | 2.31 |  |  |
| **Maximum occupational level (parents)** |  |  |  |  |  |  |  |  |  |
| Managers and Professionals |  |  |  |  |  |  |  |  |  |
| Middle level and support | 1.4 | 0.65 | 2.89 |  | 1.6 | 0.74 | 3.35 |  |  |
| Service, sales and skilled workers | 1.3 | 0.55 | 3.04 |  | 1.6 | 0.67 | 3.83 |  |  |
| Operators; unskilled & unemployed | 0.8 | 0.34 | 2.09 |  | 1.1 | 0.43 | 2.69 |  |  |
| **% of minor´s life IMSS-insured prior dx** |  |  |  |  |  |  |  |  |  |
| 80% to 100% |  |  |  |  |  |  |  |  |  |
| 50% to <80% | 1.3 | 0.74 | 2.41 |  | 1.3 | 0.72 | 2.50 |  |  |
| 25% to <50% | 2.2 | 1.18 | 4.28 | * | 2.2 | 1.15 | 4.38 | * | No change |
| < 25% | 2.4 | 1.35 | 4.42 | * | 2.6 | 1.41 | 4.74 | * | No change |
| **Distance from 3rd level hospital (km)** |  |  |  |  |  |  |  |  |  |
| < 20 |  |  |  |  |  |  |  |  |  |
| 20 to < 50 | 1.0 | 0.65 | 1.60 |  | 1.0 | 0.66 | 1.65 |  |  |
| 50 to < 200 | 0.9 | 0.52 | 1.70 |  | 0.9 | 0.52 | 1.70 |  |  |
|  200 | 0.7 | 0.25 | 1.73 |  | 1.0 | 0.39 | 2.76 |  |  |
| **Type of secondary-level hospital** |  |  |  |  |  |  |  |  |  |
| General Regional Hospital |  |  |  |  |  |  |  |  |  |
| General of Zone Hospital | 0.9 | 0.59 | 1.52 |  | 1.0 | 0.60 | 1.51 |  |  |
| General of Subzone Hospital | 2.0 | 0.55 | 7.15 |  | 1.8 | 0.50 | 6.55 |  |  |
| **Diagnosis cohort** |  |  |  |  |  |  |  |  |  |
| 2007 |  |  |  |  |  |  |  |  |  |
| 2008 | 0.7 | 0.45 | 1.19 |  | 0.6 | 0.39 | 1.03 |  |  |
| 2009 | 1.0 | 0.63 | 1.56 |  | 0.9 | 0.57 | 1.40 |  |  |

As a second step, the loss of follow-up randomness test was applied, using the follow-up losses as the dependent variable. Three variables associated with the loss were identified: Monthly family income level, distance from 3rd level hospital, and the year of diagnosis (cohort). Results are showed in table 2.

[table 2]

It is observed that three variables were associated with loss of follow-up with statistical significance: Monthly family income level, distance from 3rd level hospital and Diagnosis cohort.

**Table 2. Test of Randomness of loss to follow-up**

| **Variables** | **A. Original survival Cox model** | | | | **B. Randomness of loss to follow-up** | | | | |
| --- | --- | --- | --- | --- | --- | --- | --- | --- | --- |
|  | **Failure=Death** | | | | **Failure=Loss of follow-up** | | | | |
|  | Observations: 294, Failures: 129, P=0.000 | | | | Observations: 294, Failure: 34, P=0.0004 | | | | |
|  | **Hazard ratio** | **CI 95%** | | | **Hazard ratio** | **CI 95%** | | **p(z)** | ***** |
| **Sex** |  |  |  |  |  |  |  |  |  |
| Female |  |  |  |  |  |  |  |  |  |
| Male | 1.0 | 0.69 | 1.52 |  | 2.2 | 0.96 | 5.00 | 0.06 |  |
| **Age at ALL-diagnosis (years)** |  |  |  |  |  |  |  |  |  |
| <1 | 3.1 | 1.21 | 7.81 | * | 0.0 | 0.00 | . | 1.00 |  |
| 1 to < 5 |  |  |  |  |  |  |  |  |  |
| 5 to < 10 | 0.9 | 0.55 | 1.51 |  | 0.7 | 0.19 | 2.33 | 0.53 |  |
| 10 to <15 | 0.9 | 0.48 | 1.70 |  | 3.9 | 0.92 | 16.35 | 0.07 |  |
| **Network of services** |  |  |  |  |  |  |  |  |  |
| La Raza network |  |  |  |  |  |  |  |  |  |
| SXXI network | 0.7 | 0.31 | 1.36 |  | 1.5 | 0.36 | 5.79 | 0.60 |  |
| **ALL risk diagnosis** |  |  |  |  |  |  |  |  |  |
| Standard-risk |  |  |  |  |  |  |  |  |  |
| High-risk | 1.3 | 0.81 | 2.20 |  | 1.7 | 0.46 | 5.98 | 0.44 |  |
| **Interaction: ALL risk & network** |  |  |  |  |  |  |  |  |  |
| No high-risk dx in SXXI network |  |  |  |  |  |  |  |  |  |
| High-risk dx in SXXI network | 2.6 | 1.12 | 5.95 | * | 0.4 | 0.08 | 2.30 | 0.32 |  |
| **Monthly family income level** |  |  |  |  |  |  |  |  |  |
| 4th quartile |  |  |  |  |  |  |  |  |  |
| 3rd quartile | 1.8 | 1.00 | 3.09 |  | 0.2 | 0.07 | 0.84 | 0.03 | * |
| 2nd quartile | 1.6 | 0.90 | 2.95 |  | 0.4 | 0.13 | 1.24 | 0.11 |  |
| 1st quartile | 1.1 | 0.59 | 2.21 |  | 0.3 | 0.10 | 0.88 | 0.03 | * |
| **Availability of basic services in home** |  |  |  |  |  |  |  |  |  |
| All three present |  |  |  |  |  |  |  |  |  |
| At least one not present | 1.9 | 1.00 | 3.75 | * | 1.0 | 0.27 | 3.89 | 0.96 |  |
| **Maximum educational level (parents)** |  |  |  |  |  |  |  |  |  |
| University degree or higher |  |  |  |  |  |  |  |  |  |
| Bachelor school/ technical | 0.6 | 0.37 | 1.13 |  | 0.4 | 0.09 | 1.52 | 0.17 |  |
| High school | 0.7 | 0.35 | 1.22 |  | 1.2 | 0.29 | 5.21 | 0.78 |  |
| Primary school or less | 1.0 | 0.41 | 2.28 |  | 0.2 | 0.03 | 1.83 | 0.16 |  |
| **Maximum occupational level (parents)** |  |  |  |  |  |  |  |  |  |
| Managers and Professionals |  |  |  |  |  |  |  |  |  |
| Middle level and support | 1.4 | 0.65 | 2.89 |  | 1.4 | 0.26 | 7.93 | 0.67 |  |
| Service, sales and skilled workers | 1.3 | 0.55 | 3.04 |  | 3.3 | 0.52 | 20.30 | 0.21 |  |
| Operators; unskilled & unemployed | 0.8 | 0.34 | 2.09 |  | 2.4 | 0.34 | 17.15 | 0.37 |  |
| **% of minor´s life IMSS-insured prior dx** |  |  |  |  |  |  |  |  |  |
| 80% to 100% |  |  |  |  |  |  |  |  |  |
| 50% to <80% | 1.3 | 0.74 | 2.41 |  | 1.1 | 0.31 | 3.74 | 0.90 |  |
| 25% to <50% | 2.2 | 1.18 | 4.28 | * | 1.5 | 0.35 | 6.90 | 0.57 |  |
| < 25% | 2.4 | 1.35 | 4.42 | * | 1.5 | 0.39 | 5.56 | 0.56 |  |
| **Distance from 3rd level hospital (km)** |  |  |  |  |  |  |  |  |  |
| < 20 |  |  |  |  |  |  |  |  |  |
| 20 to < 50 | 1.0 | 0.65 | 1.60 |  | 0.9 | 0.34 | 2.25 | 0.78 |  |
| 50 to < 200 | 0.9 | 0.52 | 1.70 |  | 1.0 | 0.25 | 4.38 | 0.96 |  |
|  200 | 0.7 | 0.25 | 1.73 |  | 6.9 | 1.30 | 36.76 | 0.02 | * |
| **Type of secondary-level hospital** |  |  |  |  |  |  |  |  |  |
| General Regional Hospital |  |  |  |  |  |  |  |  |  |
| General of Zone Hospital | 0.9 | 0.59 | 1.52 |  | 1.5 | 0.44 | 4.93 | 0.53 |  |
| General of Subzone Hospital | 2.0 | 0.55 | 7.15 |  | 1.1 | 0.10 | 11.85 | 0.95 |  |
| **Diagnosis cohort** |  |  |  |  |  |  |  |  |  |
| 2007 |  |  |  |  |  |  |  |  |  |
| 2008 | 0.7 | 0.45 | 1.19 |  | 0.3 | 0.11 | 0.85 | 0.02 | * |
| 2009 | 1.0 | 0.63 | 1.56 |  | 0.4 | 0.15 | 1.04 | 0.06 |  |

On the association of the loss of follow-up and the Monthly family income level, a greater frequency of loss of follow-up was observed in children from families with higher income level (Q1: 12%, Q2: 10%, Q3: 6% and Q4 : 18%). An important fact is that the median follow-up time is 2.8 years, and there is no statistical difference in this per quartile of income, which means that the abandonment occurs during the surveillance period, and not during the treatment for all income levels. One possible reason for this is that the cost of care during the surveillance stage is much less than the cost during the treatment stages, and that the costs are more affordable for high-income families.

Regarding the association of the loss of follow-up and the distance from 3rd level hospital to the community of residence, it was observed that there is a greater frequency of loss of follow-up in children of families with habitual residence greater than 200 km from the hospital of 3rd level (<20 km: 9.2%, 20 to <50km: 12.5%, 50 to <200 km: 7%, but <200 km: 33.3%). While the median follow-up time is 3.0 years without statistical difference between distance categories less than 200 km; in children with a residence more distant than 200 km, the median follow-up is only half a year. One of the reasons for this could be the direct and indirect cost involved in transferring the child to attend their visits.

On the association of the loss of follow-up and the year of diagnosis, it was observed that there is a greater frequency of loss of follow-up in children diagnosed in 2007, this difference being statistically significant (2007: 19.4%, 2008: 6.4% and 2009: 8.3 %), with medians (2.5, 3.6 and 3.0 years respectively). These figures indicate that the quality of follow-up varies by year, and this includes both the quality of resources for active and passive case tracking.

As a next step, to evaluate the potential bias of these variables on the results we conducted a univariate sensitivity analysis for each of the 3 variables associated with the loss of follow-up.

The first scenario was defined for the variable Quartile of family income, assuming that children from families of Q3 and Q4 whose life status was unknown were alive at the end of the follow-up period; while that children of Q1 and Q2 would have died. The results of this scenario are shown in table 3.

**Table 3. Sensitivity analysis comparing original survival Cox model against scenario 1 (worst case regarding family income level)**

| **Variables** | **A. Original survival Cox model** | | | | **Scenario 1: Quartil of family income** | | | | |
| --- | --- | --- | --- | --- | --- | --- | --- | --- | --- |
|  |  |  |  |  | Loss of follow-up Q4 &Q3: alive, Q1 & Q2: dead | | | | |
|  | **Failure=Death** | | | | **Failure=Death** | | | | |
|  | Observations: 294, Failures: 129, P=0.000 | | | | Observations: 294, Failures: 146, P=0.0001 | | | | |
|  | **Hazard ratio** | **CI 95%** | | | **Hazard ratio** | **CI 95%** | | | **Changes vs A** |
| **Sex** |  |  |  |  |  |  |  |  |  |
| Female |  |  |  |  |  |  |  |  |  |
| Male | 1.0 | 0.69 | 1.52 |  | 1.1 | 0.74 | 1.55 |  |  |
| **Age at ALL-diagnosis (years)** |  |  |  |  |  |  |  |  |  |
| <1 | 3.1 | 1.21 | 7.81 | * | 2.8 | 1.15 | 7.05 | * | No change |
| 1 to < 5 |  |  |  |  |  |  |  |  |  |
| 5 to < 10 | 0.9 | 0.55 | 1.51 |  | 0.9 | 0.56 | 1.44 |  |  |
| 10 to <15 | 0.9 | 0.48 | 1.70 |  | 0.9 | 0.51 | 1.67 |  |  |
| **Network of services** |  |  |  |  |  |  |  |  |  |
| La Raza network |  |  |  |  |  |  |  |  |  |
| SXXI network | 0.7 | 0.31 | 1.36 |  | 0.9 | 0.46 | 1.67 |  |  |
| **ALL risk diagnosis** |  |  |  |  |  |  |  |  |  |
| Standard-risk |  |  |  |  |  |  |  |  |  |
| High-risk | 1.3 | 0.81 | 2.20 |  | 1.4 | 0.90 | 2.31 |  |  |
| **Interaction: ALL risk & network** |  |  |  |  |  |  |  |  |  |
| No high-risk dx in SXXI network |  |  |  |  |  |  |  |  |  |
| High-risk dx in SXXI network | 2.6 | 1.12 | 5.95 | * | 1.8 | 0.86 | 3.84 |  | Lost significance |
| **Monthly family income level** |  |  |  |  |  |  |  |  |  |
| 4th quartile |  |  |  |  |  |  |  |  |  |
| 3rd quartile | 1.8 | 1.00 | 3.09 |  | 1.8 | 1.01 | 3.12 | * | Gain significance |
| 2nd quartile | 1.6 | 0.90 | 2.95 |  | 2.0 | 1.13 | 3.57 | * | Gain significance |
| 1st quartile | 1.1 | 0.59 | 2.21 |  | 1.4 | 0.77 | 2.66 |  |  |
| **Availability of basic services in home** |  |  |  |  |  |  |  |  |  |
| All three present |  |  |  |  |  |  |  |  |  |
| At least one not present | 1.9 | 1.00 | 3.75 | * | 1.7 | 0.90 | 3.20 |  | Lost significance |
| **Maximum educational level (parents)** |  |  |  |  |  |  |  |  |  |
| University degree or higher |  |  |  |  |  |  |  |  |  |
| Bachelor school/ technical | 0.6 | 0.37 | 1.13 |  | 0.6 | 0.34 | 1.00 |  |  |
| High school | 0.7 | 0.35 | 1.22 |  | 0.7 | 0.38 | 1.24 |  |  |
| Primary school or less | 1.0 | 0.41 | 2.28 |  | 0.8 | 0.36 | 1.85 |  |  |
| **Maximum occupational level (parents)** |  |  |  |  |  |  |  |  |  |
| Managers and Professionals |  |  |  |  |  |  |  |  |  |
| Middle level and support | 1.4 | 0.65 | 2.89 |  | 1.3 | 0.63 | 2.86 |  |  |
| Service, sales and skilled workers | 1.3 | 0.55 | 3.04 |  | 1.5 | 0.63 | 3.40 |  |  |
| Operators; unskilled & unemployed | 0.8 | 0.34 | 2.09 |  | 0.9 | 0.39 | 2.31 |  |  |
| **% of minor´s life IMSS-insured prior dx** |  |  |  |  |  |  |  |  |  |
| 80% to 100% |  |  |  |  |  |  |  |  |  |
| 50% to <80% | 1.3 | 0.74 | 2.41 |  | 1.3 | 0.76 | 2.31 |  |  |
| 25% to <50% | 2.2 | 1.18 | 4.28 | * | 2.3 | 1.26 | 4.17 | * | No change |
| < 25% | 2.4 | 1.35 | 4.42 | * | 2.2 | 1.27 | 3.82 | * | No change |
| **Distance from 3rd level hospital (km)** |  |  |  |  |  |  |  |  |  |
| < 20 |  |  |  |  |  |  |  |  |  |
| 20 to < 50 | 1.0 | 0.65 | 1.60 |  | 1.0 | 0.68 | 1.59 |  |  |
| 50 to < 200 | 0.9 | 0.52 | 1.70 |  | 1.0 | 0.55 | 1.67 |  |  |
|  200 | 0.7 | 0.25 | 1.73 |  | 1.0 | 0.44 | 2.32 |  |  |
| **Type of secondary-level hospital** |  |  |  |  |  |  |  |  |  |
| General Regional Hospital |  |  |  |  |  |  |  |  |  |
| General of Zone Hospital | 0.9 | 0.59 | 1.52 |  | 1.0 | 0.62 | 1.51 |  |  |
| General of Subzone Hospital | 2.0 | 0.55 | 7.15 |  | 1.6 | 0.51 | 4.90 |  |  |
| **Diagnosis cohort** |  |  |  |  |  |  |  |  |  |
| 2007 |  |  |  |  |  |  |  |  |  |
| 2008 | 0.7 | 0.45 | 1.19 |  | 0.7 | 0.42 | 1.03 |  |  |
| 2009 | 1.0 | 0.63 | 1.56 |  | 0.8 | 0.55 | 1.29 |  |  |

The second scenario was defined for the variable Distance from hospital tertiary, assuming that children with a residence less than 200 km whose life status was unknown, would be alive at the end of the follow-up period; while children with residence greater than 200 km would have died. The results of this model are shown in table 4.

[table 4]

**Table 4. Sensitivity analysis comparing original survival Cox model against scenario 2 (worst case regarding Distance from tertiary hospital)**

| **Variables** | **A. Original survival Cox model** | | | | **Scenario 2: Distance from tertiary hospital** | | | | |
| --- | --- | --- | --- | --- | --- | --- | --- | --- | --- |
|  |  |  |  |  | Loss of follow-up closer than 200km: alive, farther: dead | | | | |
|  | **Failure= Death** | | | | **Failure= Death** | | | | |
|  | Observations: 294, Failures: 129, P=0.000 | | | | Observations: 294, Failures: 136, P=0.0001 | | | | |
|  | **Hazard ratio** | **CI 95%** | | | **Hazard ratio** | **CI 95%** | | | **Changes vs A** |
| **Sex** |  |  |  |  |  |  |  |  |  |
| Female |  |  |  |  |  |  |  |  |  |
| Male | 1.0 | 0.69 | 1.52 |  | 1.1 | 0.74 | 1.60 |  |  |
| **Age at ALL-diagnosis (years)** |  |  |  |  |  |  |  |  |  |
| <1 | 3.1 | 1.21 | 7.81 | * | 2.7 | 1.09 | 6.91 | * | No change |
| 1 to < 5 |  |  |  |  |  |  |  |  |  |
| 5 to < 10 | 0.9 | 0.55 | 1.51 |  | 1.0 | 0.58 | 1.56 |  |  |
| 10 to <15 | 0.9 | 0.48 | 1.70 |  | 0.9 | 0.50 | 1.72 |  |  |
| **Network of services** |  |  |  |  |  |  |  |  |  |
| La Raza network |  |  |  |  |  |  |  |  |  |
| SXXI network | 0.7 | 0.31 | 1.36 |  | 0.8 | 0.43 | 1.64 |  |  |
| **ALL risk diagnosis** |  |  |  |  |  |  |  |  |  |
| Standard-risk |  |  |  |  |  |  |  |  |  |
| High-risk | 1.3 | 0.81 | 2.20 |  | 1.5 | 0.92 | 2.47 |  |  |
| **Interaction: ALL risk & network** |  |  |  |  |  |  |  |  |  |
| No high-risk dx in SXXI network |  |  |  |  |  |  |  |  |  |
| High-risk dx in SXXI network | 2.6 | 1.12 | 5.95 | * | 1.8 | 0.84 | 3.91 |  | Lost significance |
| **Monthly family income level** |  |  |  |  |  |  |  |  |  |
| 4th quartile |  |  |  |  |  |  |  |  |  |
| 3rd quartile | 1.8 | 1.00 | 3.09 |  | 1.6 | 0.94 | 2.81 |  |  |
| 2nd quartile | 1.6 | 0.90 | 2.95 |  | 1.5 | 0.87 | 2.70 |  |  |
| 1st quartile | 1.1 | 0.59 | 2.21 |  | 1.1 | 0.58 | 2.01 |  |  |
| **Availability of basic services in home** |  |  |  |  |  |  |  |  |  |
| All three present |  |  |  |  |  |  |  |  |  |
| At least one not present | 1.9 | 1.00 | 3.75 | * | 1.8 | 0.93 | 3.30 |  | Lost significance |
| **Maximum educational level (parents)** |  |  |  |  |  |  |  |  |  |
| University degree or higher |  |  |  |  |  |  |  |  |  |
| Bachelor school/ technical | 0.6 | 0.37 | 1.13 |  | 0.7 | 0.39 | 1.15 |  |  |
| High school | 0.7 | 0.35 | 1.22 |  | 0.7 | 0.37 | 1.26 |  |  |
| Primary school or less | 1.0 | 0.41 | 2.28 |  | 1.0 | 0.42 | 2.29 |  |  |
| **Maximum occupational level (parents)** |  |  |  |  |  |  |  |  |  |
| Managers and Professionals |  |  |  |  |  |  |  |  |  |
| Middle level and support | 1.4 | 0.65 | 2.89 |  | 1.4 | 0.65 | 2.84 |  |  |
| Service, sales and skilled workers | 1.3 | 0.55 | 3.04 |  | 1.5 | 0.64 | 3.38 |  |  |
| Operators; unskilled & unemployed | 0.8 | 0.34 | 2.09 |  | 0.9 | 0.37 | 2.18 |  |  |
| **% of minor´s life IMSS-insured prior dx** |  |  |  |  |  |  |  |  |  |
| 80% to 100% |  |  |  |  |  |  |  |  |  |
| 50% to <80% | 1.3 | 0.74 | 2.41 |  | 1.2 | 0.68 | 2.19 |  |  |
| 25% to <50% | 2.2 | 1.18 | 4.28 | * | 2.1 | 1.11 | 3.95 | * | No change |
| < 25% | 2.4 | 1.35 | 4.42 | * | 2.3 | 1.28 | 4.00 | * | No change |
| **Distance from 3rd level hospital (km)** |  |  |  |  |  |  |  |  |  |
| < 20 |  |  |  |  |  |  |  |  |  |
| 20 to < 50 | 1.0 | 0.65 | 1.60 |  | 1.0 | 0.63 | 1.55 |  |  |
| 50 to < 200 | 0.9 | 0.52 | 1.70 |  | 1.0 | 0.56 | 1.80 |  |  |
|  200 | 0.7 | 0.25 | 1.73 |  | 1.5 | 0.68 | 3.36 |  |  |
| **Type of secondary-level hospital** |  |  |  |  |  |  |  |  |  |
| General Regional Hospital |  |  |  |  |  |  |  |  |  |
| General of Zone Hospital | 0.9 | 0.59 | 1.52 |  | 1.0 | 0.65 | 1.68 |  |  |
| General of Subzone Hospital | 2.0 | 0.55 | 7.15 |  | 1.6 | 0.50 | 5.05 |  |  |
| **Diagnosis cohort** |  |  |  |  |  |  |  |  |  |
| 2007 |  |  |  |  |  |  |  |  |  |
| 2008 | 0.7 | 0.45 | 1.19 |  | 0.7 | 0.42 | 1.10 |  |  |
| 2009 | 1.0 | 0.63 | 1.56 |  | 0.9 | 0.60 | 1.46 |  |  |

The third scenario was defined for the Diagnosis cohort variable, assuming that children diagnosed during 2008 and 2009 whose life status was unknown, would be alive at the end of the follow-up period; while children diagnosed in 2007 would have died. The results of this model are shown in table 5.

[table 5]

**Table 5. Sensitivity analysis comparing original survival Cox model against scenario 3 (worst case regarding diagnosis cohort)**

| **Variables** | **A. Original survival Cox model** | | | | **Scenario 3: Diagnosis cohort** | | | | |
| --- | --- | --- | --- | --- | --- | --- | --- | --- | --- |
|  |  |  |  |  | Loss of follow-up 2008&09: alive, 2007: dead | | | | |
|  | **Failure= Death** | | | | **Failure= Death** | | | | |
|  | Observations: 294, Failures: 129, P=0.000 | | | | Observations: 294, Failures: 149, P=0.0000 | | | | |
|  | **Hazard ratio** | **CI 95%** | | | **Hazard ratio** | **CI 95%** | | | **Changes vs A** |
| **Sex** |  |  |  |  |  |  |  |  |  |
| Female |  |  |  |  |  |  |  |  |  |
| Male | 1.0 | 0.69 | 1.52 |  | 1.2 | 0.83 | 1.73 |  |  |
| **Age at ALL-diagnosis (years)** |  |  |  |  |  |  |  |  |  |
| <1 | 3.1 | 1.21 | 7.81 | * | 3.0 | 1.21 | 7.46 | * | No change |
| 1 to < 5 |  |  |  |  |  |  |  |  |  |
| 5 to < 10 | 0.9 | 0.55 | 1.51 |  | 1.0 | 0.60 | 1.56 |  |  |
| 10 to <15 | 0.9 | 0.48 | 1.70 |  | 1.1 | 0.59 | 1.95 |  |  |
| **Network of services** |  |  |  |  |  |  |  |  |  |
| La Raza network |  |  |  |  |  |  |  |  |  |
| SXXI network | 0.7 | 0.31 | 1.36 |  | 0.7 | 0.37 | 1.40 |  |  |
| **ALL risk diagnosis** |  |  |  |  |  |  |  |  |  |
| Standard-risk |  |  |  |  |  |  |  |  |  |
| High-risk | 1.3 | 0.81 | 2.20 |  | 1.4 | 0.88 | 2.29 |  |  |
| **Interaction: ALL risk & network** |  |  |  |  |  |  |  |  |  |
| No high-risk dx in SXXI network |  |  |  |  |  |  |  |  |  |
| High-risk dx in SXXI network | 2.6 | 1.12 | 5.95 | * | 2.0 | 0.94 | 4.29 |  | Lost significance |
| **Monthly family income level** |  |  |  |  |  |  |  |  |  |
| 4th quartile |  |  |  |  |  |  |  |  |  |
| 3rd quartile | 1.8 | 1.00 | 3.09 |  | 1.4 | 0.82 | 2.32 |  |  |
| 2nd quartile | 1.6 | 0.90 | 2.95 |  | 1.4 | 0.85 | 2.46 |  |  |
| 1st quartile | 1.1 | 0.59 | 2.21 |  | 1.0 | 0.54 | 1.73 |  |  |
| **Availability of basic services in home** |  |  |  |  |  |  |  |  |  |
| All three present |  |  |  |  |  |  |  |  |  |
| At least one not present | 1.9 | 1.00 | 3.75 | * | 1.8 | 0.97 | 3.22 |  | Lost significance |
| **Maximum educational level (parents)** |  |  |  |  |  |  |  |  |  |
| University degree or higher |  |  |  |  |  |  |  |  |  |
| Bachelor school/ technical | 0.6 | 0.37 | 1.13 |  | 0.6 | 0.36 | 1.05 |  |  |
| High school | 0.7 | 0.35 | 1.22 |  | 0.6 | 0.36 | 1.16 |  |  |
| Primary school or less | 1.0 | 0.41 | 2.28 |  | 0.8 | 0.38 | 1.85 |  |  |
| **Maximum occupational level (parents)** |  |  |  |  |  |  |  |  |  |
| Managers and Professionals |  |  |  |  |  |  |  |  |  |
| Middle level and support | 1.4 | 0.65 | 2.89 |  | 1.2 | 0.59 | 2.39 |  |  |
| Service, sales and skilled workers | 1.3 | 0.55 | 3.04 |  | 1.3 | 0.61 | 2.97 |  |  |
| Operators; unskilled & unemployed | 0.8 | 0.34 | 2.09 |  | 0.9 | 0.38 | 2.03 |  |  |
| **% of minor´s life IMSS-insured prior dx** |  |  |  |  |  |  |  |  |  |
| 80% to 100% |  |  |  |  |  |  |  |  |  |
| 50% to <80% | 1.3 | 0.74 | 2.41 |  | 1.4 | 0.79 | 2.37 |  |  |
| 25% to <50% | 2.2 | 1.18 | 4.28 | * | 2.1 | 1.13 | 3.90 | * | No change |
| < 25% | 2.4 | 1.35 | 4.42 | * | 2.2 | 1.27 | 3.85 | * | No change |
| **Distance from 3rd level hospital (km)** |  |  |  |  |  |  |  |  |  |
| < 20 |  |  |  |  |  |  |  |  |  |
| 20 to < 50 | 1.0 | 0.65 | 1.60 |  | 1.0 | 0.66 | 1.55 |  |  |
| 50 to < 200 | 0.9 | 0.52 | 1.70 |  | 1.0 | 0.57 | 1.73 |  |  |
|  200 | 0.7 | 0.25 | 1.73 |  | 1.1 | 0.50 | 2.55 |  |  |
| **Type of secondary-level hospital** |  |  |  |  |  |  |  |  |  |
| General Regional Hospital |  |  |  |  |  |  |  |  |  |
| General of Zone Hospital | 0.9 | 0.59 | 1.52 |  | 1.1 | 0.67 | 1.67 |  |  |
| General of Subzone Hospital | 2.0 | 0.55 | 7.15 |  | 1.8 | 0.58 | 5.54 |  |  |
| **Diagnosis cohort** |  |  |  |  |  |  |  |  |  |
| 2007 |  |  |  |  |  |  |  |  |  |
| 2008 | 0.7 | 0.45 | 1.19 |  | 0.5 | 0.32 | 0.80 | * | Gain significance |
| 2009 | 1.0 | 0.63 | 1.56 |  | 0.7 | 0.45 | 1.05 |  |  |

Of the 3 scenarios, shown in tables 3 to 5, it is observed that the effect of two variables is not stable in relation to the original model: Interaction: ALL diagnosis risk & network of medical services; and the variable Availability of basic services in home. However, inconsistencies were not generated in the direction of the coefficients of these variables or others in the model. So we decided not to take as robust the effect of these variables.

On the other hand, the effect of the variables age and proportion of children's life IMSS-insured prior ALL-diagnosis were maintained in the 3 scenarios in direction, trend and magnitude. Therefore, it was decided to consider the results of the original model for this variable robust.
